# First real-world clinical experience with [^177^Lu]Lu-PSMA-I&T in patients with metastatic castration-resistant prostate cancer beyond VISION and TheraP criteria

**DOI:** 10.1007/s00259-025-07082-9

**Published:** 2025-01-18

**Authors:** Sui wai Ling, Quido de Lussanet de la Sablonière, Michael Ananta, Erik de Blois, Stijn L. W. Koolen, Roosmarijn C. Drexhage, Johannes Hofland, Debbie G. J. Robbrecht, Astrid A M van der Veldt, Frederik A Verburg, Tessa Brabander

**Affiliations:** 1 Department of Radiology & Nuclear Medicine, Erasmus MC, Rotterdam, The Netherlands; 2 Department of Hospital Pharmacy, Erasmus MC, Rotterdam, The Netherlands; 3 Department of Medical Oncology, Erasmus MC Cancer Institute, Rotterdam, The Netherlands; 4 Department of Internal Medicine, Section of Endocrinology, Erasmus MC Cancer Institute, Rotterdam, The Netherlands

**Keywords:** PSMA, Prostate cancer, Lutetium-177, Radionuclide therapy

## Abstract

**Purpose:**

To report real-world clinical experience with [^177^Lu]Lu-PSMA-I&T targeted radionuclide therapy (TRT) in patients with metastatic castration-resistant prostate cancer (mCRPC) in a single tertiary referral university hospital.

**Methods:**

Patients with mCRPC who were treated with [^177^Lu]Lu-PSMA-I&T TRT as standard of care between February 2022 and August 2023 were included in this retrospective study. Patients were treated with a maximum of six cycles with a fixed activity of 7.4 GBq/100µg [^177^Lu]Lu-PSMA-I&T per cycle.

**Results:**

50 patients with mCRPC were included, of them 84% had prior therapy with two lines of taxane-based chemotherapy treated and at least one line of androgen receptor signaling inhibitor. A total of 126 cycles with a median of 2 cycles (IQR 1–6) [ ^177^Lu]Lu-PSMA-I&T were administered per patient. PSA declines of ≥ 50% and ≥ 70% were achieved in 16% and 10% of the patients, respectively. Radiological response was achieved in 11% of the patients. In total, 68 treatment-related Adverse Events (TRAEs) were observed, mainly grade 1–2 in 88% of cases. Grade 3/4 TRAEs were observed in 12% of cases. No grade 3 or higher xerostomia was reported. Median progression-free survival was 7.7 months (95% CI 4.0-11.3) and median overall survival was 8.1 months (95% CI 5.0-11.3).

**Conclusion:**

In heavily pretreated patients with mCRPC, treatment of [^177^Lu]Lu-PSMA-I&T TRT is well tolerated and safe, but real-world efficacy of [^177^Lu]Lu-PSMA appears lower compared to data from recent phase-3 clinical trials using a different radioligand [^177^Lu]Lu-PSMA-617. Further studies may show whether patients with mCRPC benefit more from [^177^Lu]Lu-PSMA when initiated at an earlier stage of treatment.

## Introduction

After primary diagnosis of prostate cancer, approximately 10–20% of patients develop castration-resistant prostate cancer (CRPC) and most (> 80%) of these patients have advanced or metastatic disease (mCRPC) [1]. Although systemic treatments such as androgen receptor signaling inhibitors (ARSI), taxane-based chemotherapies and Radium-223 are available for patients with mCRPC, survival benefit is limited to a median of 3–4 months for every treatment line [2, 3]. Therefore, there is a need for new treatments to improve patient outcomes.

In the phase III VISION [4] and phase II TheraP [5] trials, efficacy of [^177^Lu]Lu-PSMA-617 was shown in patients with mCRPC who were previously treated with androgen receptor inhibitors and at least one line of taxane-based chemotherapy. As a result, the Food and Drug Administration and European Medicines Agency (EMA) approved [^177^Lu]Lu-PSMA-617 for this patient population in 2022.

While awaiting reimbursement of [^177^Lu]Lu-PSMA-617 in the Netherlands, [^177^Lu]Lu-PSMA-Imaging & Therapy (I&T) is available for patients who would be otherwise eligible for [^177^Lu]Lu-PSMA-617 according to the EMA label [6]. However, the two PSMA compounds show chemical structural differences. Whilst the urea-based binding motifs are identical, the chelators are significantly different: [^177^Lu]Lu-PSMA-617 is linked to a DOTA-chelator and [^177^Lu]Lu-PSMA-I&T is linked to a DOTAGA-chelator [7]. Nonetheless, the joint EANM/SNMMI procedure guideline consider PSMA-617 and PSMA-I&T as equivalent for PSMA TRT [8]. Previous preclinical studies have demonstrated comparable in vivo behavior between PSMA-I&T and PSMA-617 [9]. Also, the two PSMA ligands showed comparable toxicity and efficacy profile in patients with mCRPC [7, 10]. Here, we report our first real-world clinical experience with [^177^Lu]Lu-PSMA-I&T targeted radionuclide therapy (TRT) in patients with mCRPC.

## Materials and methods

### Study design

This single center retrospective study was approved by the institutional ethics board (MEC-2024-0104). All consecutive patients with mCRPC who were treated between February 2022 and August 2023 with [^177^Lu]Lu-PSMA-I&T TRT as part of the standard clinical care were included.

### Objectives

The primary objective of this study was to investigate the efficacy of [^177^Lu]Lu-PSMA-I&T TRT in patients with mCRPC assessed by the best overall PSA response, radiographic response (defined by RECIST 1.1) and survival outcomes (biochemical and radiographic PFS and OS). The secondary objective of this study was to investigate the safety of [^177^Lu]Lu-PSMA-I&T as assessed by the incidence and severity of the (serious) adverse events ((S)AEs) for the dose-limiting organs.

### Eligibility

Local eligibility criteria for [^177^Lu]Lu-PSMA-I&T TRT were Eastern Cooperative Oncology Group (ECOG) performance status (PS) of 0–2, at least one line of ARSI and two line of taxane-based chemotherapy. Except for patients who had absolute contra-indications for taxane-based chemotherapy or who did not wish to receive taxane-based chemotherapy were also eligible for treatment with [^177^Lu]Lu-PSMA-I&T. Patients were included if they had sufficient uptake on [^68^Ga]Ga-PSMA-11 PET-CT according to the criteria described in the VISION trial [4]. Other inclusion criteria were serum hemoglobin  ≥ 5.5 mmol/L, total white blood cell count  ≥ 3 × 10^9^/L, platelet count  ≥ 100 × 10^9^/L and creatinine clearance  ≥ 30 mL/minute.

### Treatment

[^177^Lu]Lu-PSMA-I&T was produced in-house in a compassionate use program, approved by the Dutch health authority for patients with mCRPC and no other anti-cancer treatment options [11]. [^177^Lu]Lu-PSMA-I&T TRT consisted of four cycles with a fixed activity of 7.4 GBq (100 µg PSMA-I&T) per cycle [11]. Patients with clinical benefit after four cycles could be treated with two additional cycles. An 8-week (range 6–12) interval between consecutive cycles was chosen based on our experience with peptide receptor radionuclide therapy (i.e. [^177^Lu]Lu-DOTATATE) at the Erasmus MC [12, 13]. [^177^Lu]Lu-PSMA-I&T was administered intravenously using a infusion pump with an infusion speed of 200 mL/hour. Cold packs (approx. 4 °C) were applied around the salivary glands from 30 min before, until four hours after administration of [^177^Lu]Lu-PSMA-I&T and were changed every 30 min.

Safety visits and biochemical response evaluations were performed five weeks after each cycle and radiographic response evaluation with contrast enhanced CT was performed after every two cycles according to RECIST 1.1 until progression [14].

### Data collection

Data were retrieved from the electronic patient records. AEs and SAEs according to Common Terminology Criteria for Adverse Events version 5 were collected for the dose-limiting organs (i.e. salivary glands, kidney and bone marrow) [15].

### Statistical analysis

All continuous data were reported as descriptive values. Progression-free survival (PFS) was defined as the interval between the first cycle of [^177^Lu]Lu-PSMA-I&T and disease progression (radiological progression according to RECIST 1.1 and/or clinical/biochemical progression) or death from any cause. Overall survival (OS) was defined as the interval between the first cycle of [^177^Lu]Lu-PSMA-I&T and death from any cause. For the calculation of PFS and OS the Kaplan-Meier analysis was used. Statistical analyses were performed using SPSS (version 28.0.1.0, IBM Corp., Armonk, NY, USA).

## Results

Fifty patients with mCRPC were included. ECOG PS 0–1 and PS 2 were reported in 40 (80%) and 7 (14%) patients, respectively, while ECOG PS was missing in 3 (6%) patients. Metastases was observed in 100% (bones), 66% (lymph nodes), 20% (adrenal glands), 14% (liver) and 12% (lungs) of the patients. Most of the patients (84%) were treated with multiple prior lines consisting of at least one line of ARSI and two lines of taxane-based chemotherapy. Baseline characteristics are summarized in Table 1.

**Table 1:** Baseline characteristics and studies presented for comparison. ADT = androgen deprivation therapy. ARSI = androgen receptor signaling inhibitor. EBRT = External Beam Radiotherapy. NR = not reported. ECOG = Eastern Cooperative Oncology Group

Baseline		Total (*N* = 50)	VISION (*N* = 551)	TheraP (*N* = 99)
		Median (IQR)	Median (IQR)	Median (IQR)
Age		70 (64–75)	70 (48–94)	72 (67–77)
		N (%)	N (%)	N (%)
ECOG	0	4 (8)	NR	42 (42)
	1	36 (72)	NR	53 (54)
	0–1	40 (80)	510 (93)	95 (96)
	2	7 (14)	41 (7)	4 (4)
	NR	3 (6)	0	0
Metastatic sites	Bone	50 (100)	504 (92)	90 (91)
	Lymph nodes	33 (66)	274 (50)	52 (53)
	Liver	7 (14)	63 (11)	NR
	Lung	6 (12)	49 (9)	NR
	Adrenal glands	10 (20)	NR	NR
Previous therapies	Prostatectomy	13 (26)	240 (44)	43 (43)
	Radiotherapy	30 (60)	415 (75)	40 (40)
	ADT	48 (96)	NR	NR
	** Chemotherapy**			
	Docetaxel	45 (90)	534 (97)	NR
	Cabazitaxel	44 (88)	209 (38)	0
	One regimen	4 (8)	325 (59)	
	Two regimens	42 (84)	220 (40)	
	** ARSI**			
	Enzalutamide	34 (68)	395 (72)	49 (50)
	Abiraterone	22 (44)	187 (34)	21 (21)
	Apalutamide	3 (6)	13 (2)	NR
	One regimen	42 (84)	298 (54)	70 (71)
	Two regimens	7 (14)	213 (39)	21(21)
	> 2 regimens	1 (2)	40 (7)	NR
	Radium-223	14 (28)	NR	NR
	^225^Ac-PSMA I&T	2 (4)	NR	NR

A total of 126 cycles of [^177^Lu]Lu-PSMA-I&T with a median of two cycles (IQR 1–6) per patient were administered. One patient previously received two cycles of [^177^Lu]Lu-PSMA-617 as first-line treatment in the hormone-sensitive phase elsewhere, approximately 13 years prior to treatment with [^177^Lu]Lu-PSMA-I&T in our center.

Overall, a PSA decrease of ≥ 50% was measured in 8 (16%) of 50 patients and a PSA decrease of ≥ 70% was measured in 5 (10%) patients. According to RECIST 1.1, 28 patients had evaluable disease. Radiological response was observed in 3 (11%) of 28 patients (Table 2).

**Table 2:** Response evaluation. PSA = prostate specific Antigen. PR = partial response. RECIST = response evaluation criteria in solid tumors. SD = stable disease

Responses	Our study *N* (%)	VISION *N*(%)	TheraP *N* (%)
Biochemical			
Total patients	*n* = 50	*n* = 385	*n* = 98
PSA ≥ 50%	8 (16)	177 (46)	65 (66)
Radiological			
Total patients	*n* = 28	*n* = 184	*n* = 37
Response Rate (RECIST)	3 (11)	95 (51.5)	18 (49)

**Fig. 1:**
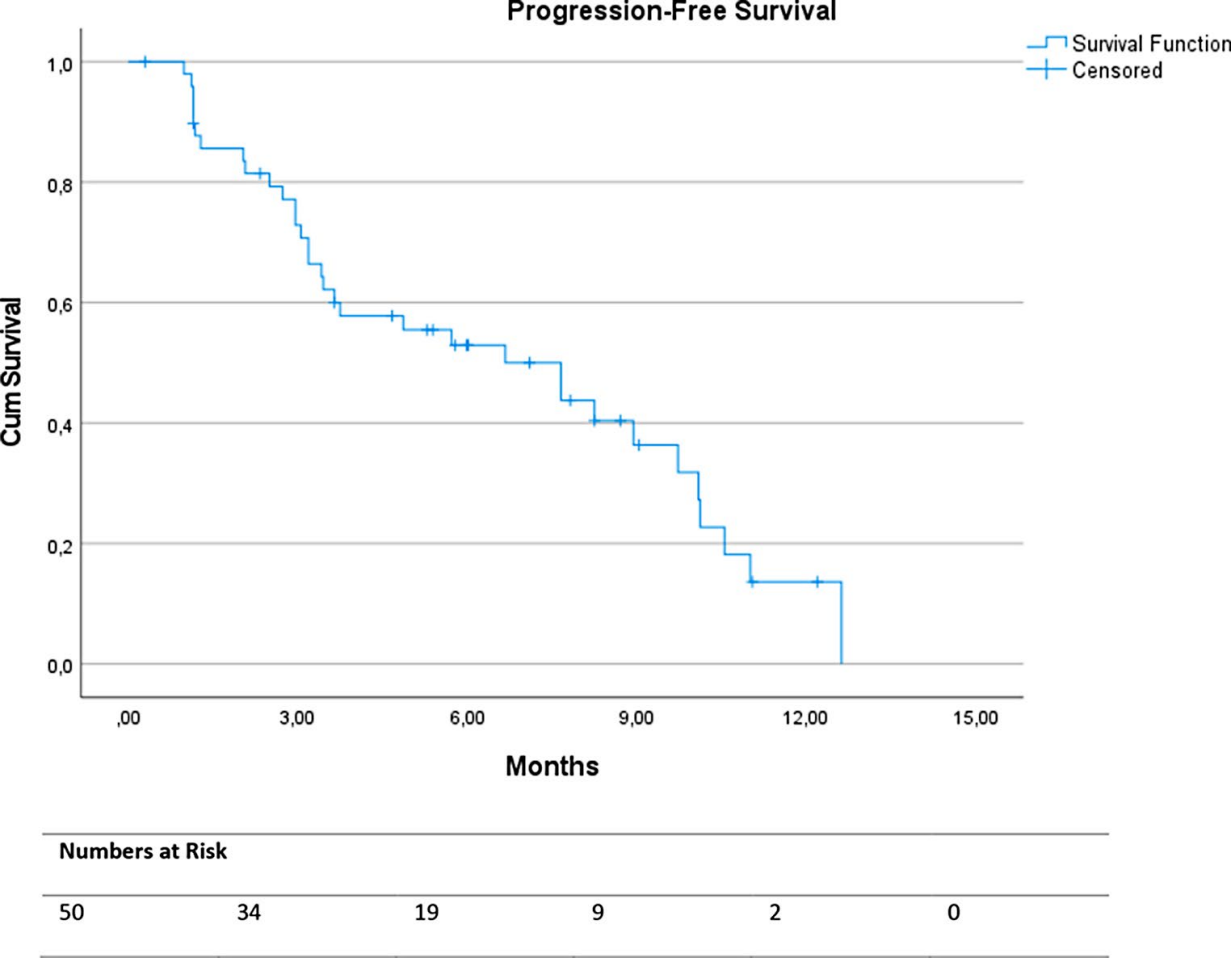
Progression-free survival in months

**Fig. 2:**
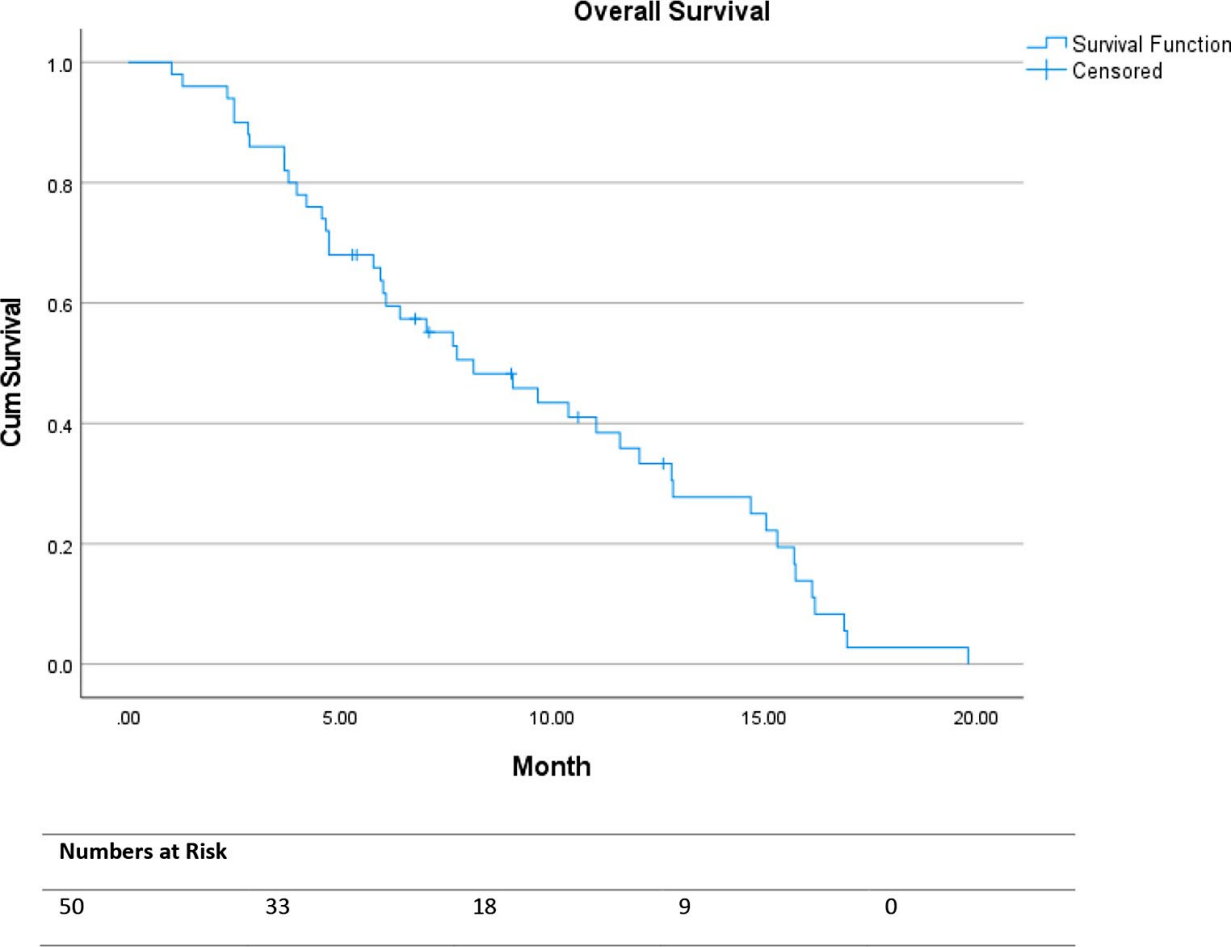
Overall survival in months

Treatment was discontinued after 1 or 2 cycles in 28 (56%) of the patients due to progressive disease (*n* = 24) or adverse events (*n* = 3). The median PFS was 7.7 months (95% CI 4.0-11.3) and the median OS was 8.1 months (95% CI 5.0-11.3; see Figs. 1 and 2).

Most of the AEs were limited to grade 1 and 2. Grade 1–2 AEs included xerostomia (46%), increased creatinine (10%), anemia (20%), thrombocytopenia (28%) and leukocytopenia (16%). More severe AEs included grade 3 anemia (14%) and grade 4 thrombocytopenia (2%) An overview of all AEs is presented in Table 3.

## Discussion

In this heavily pretreated cohort of mCRPC patients, [^177^Lu]Lu-PSMA-I&T TRT was tolerated well, but showed moderate efficacy in terms of PSA, radiological responses and OS. Nevertheless, PFS appeared comparable to previous studies [16].

Compared to the imaging response rates (based on RECIST v1.1.) from the VISION (51.5%) and TheraP (49%) trials (in which a different radioligand ([^177^Lu]Lu-PSMA-617) was used) [4, 5], the therapeutic effect was less in our patient cohort (11%), which may be explained by the difference in patient characteristics. In both the VISION trial and TheraP trial, patients had less previous treatments, and in the TheraP trial patients were randomized between [^177^Lu]Lu-PSMA-617 and cabazitaxel. In the [^177^Lu]Lu-PSMA-617 arm of the VISION trial, 38% of patients was pretreated with cabazitaxel in second or third line setting, compared to 84% of patients in our cohort (Table 3).

**Table 3:** Frequency of adverse events after [^177^Lu]Lu-PSMA-I&T radionuclide therapy. NR = not reported

Adverse events	Our study		VISION		TheraP	
Total patients	*n* = 50		*n* = 529		*n* = 98	
	Grade 1–2 *N* (%)	Grade 3–4 *N* (%)	Grade 1–2 *N* (%)	Grade 3–4 *N* (%)	Grade 1–2 *N* (%)	Grade 3–4 *N* (%)
Xerostomia	23 (46)	-	205 (39)	-	59 (60)	-
Creatinine Increased	5 (10)	-	28 (5.3)	1 (0.2)	4 (4)	-
Anemia	10 (20)	7 (14)	100 (19)	68 (12.9)	19 (19)	8 (8)
Thrombocytopenia	14 (28)	1 (2)	49 (9)	42 (8)	18 (18)	11 (11)
Leukocytopenia	8 (16)	-	54 (10)	12 (2.5)	10 (10)	1 (1)

Although the patients in our cohort had more previous treatments, limited objective tumor responses, and a lower median number of cycles of treatment, the PFS (7.7 months) was comparable to the PFS (8.7 months) as reported in the VISION trial, however the OS (8.1 months) was lower compared to the OS (15.3 months) of the VISION trial [4]. Recently, the U.S. Expanded Access Program showed a median OS of 13.7 months with [^177^Lu]Lu-PSMA-617 TRT in patients with mCRPC who were more heavily pretreated with ARSIs [17].

In general, we observed a higher frequency of AEs as compared to both the VISION and TheraP trials, and in particular, more hematological AEs were reported in our cohort. Since more than 80% of patients were previously treated with at least one line of taxanes, their bone marrow capacity was probably already more affected prior to the administration of [^177^Lu]Lu-PSMA-I&T. Studies have shown that previous chemotherapy and extensive bone involvement could be considered as risk factors for increased myelotoxicity with ^177^Lu-PSMA TRT [18–21].

Finally, the responses of [^177^Lu]Lu-PSMA TRT in patients with late stage mCRPC is limited and fuels thoughts for improvement. This limited efficacy may be caused by the increased PSMA heterogeneity in later stages of the disease, which is also influenced by previous anti-tumor therapies [22]. Therefore, treatment with [^177^Lu]Lu-PSMA in earlier disease stages of mCRPC is currently under investigation [23]. A small group of patients with metastatic hormone-sensitive prostate cancer (mHSPC) who were treated with [^177^Lu]Lu-PSMA-I&T showed promising PSA responses [24]. Other studies, such as the Bullseye trial (NCT04443062), PSMAddition trial (NCT04720157) and UpfrontPSMA trial (NCT04343885), are currently ongoing to assess the clinical impact of [^177^Lu]Lu-PSMA TRT in patients with mHSPC. Furthermore, a correlation between treatment response and health-related quality of life was found in mCRPC patients treated with [^177^Lu]Lu-PSMA-I&T [25]. Also, strategies to improve survival outcomes have been investigated in retrospective studies by identifying clinical baseline characteristics or biomarkers in patients with mCRPC who were treated with [^177^Lu]Lu-PSMA-I&T [26, 27]. Lastly, preliminary results of the phase 3 SPLASH study (NCT04647526) showed an improved radiographic PFS and PSA response in patients with mCRPC who were treated with [^177^Lu]Lu-PSMA-I&T.

This study has several limitations. First, the relatively small sample size may have influenced our results of PFS and OS. Second, other clinical responses (such as pain evaluation) were not assessed. Third, this is a retrospective study, so the results are affected by a non-negligible bias. Fourth, a different radiopharmaceutical was used from the trials referred to; despite the two radiopharmaceuticals being treated as equivalent, differences in efficacy have not been proven yet. Last, included patients had different inclusion criteria than the referred trials, especially from TheraP, so the significance of the comparison might result in misleading outcomes.

## Conclusions

In heavily pretreated patients with mCRPC, treatment of [^177^Lu]Lu-PSMA-I&T TRT is well tolerated and safe, but real-world efficacy of [^177^Lu]Lu-PSMA appears lower compared to data from recent phase-3 clinical trials using a different radioligand [^177^Lu]Lu-PSMA-617. These findings highlight the need for adequate patient selection and the evaluation of [^177^Lu]Lu-PSMA in earlier disease stages.

## Data Availability

The datasets generated during and/or analysed during the current study are available from the corresponding author on reasonable request.
